# Regulation of hippocampal neuronal apoptosis and autophagy in mice with sepsis‐associated encephalopathy by immunity‐related GTPase M1

**DOI:** 10.1111/cns.13229

**Published:** 2019-10-14

**Authors:** Rui‐Xi Zhou, Yu‐Yao Li, Yi Qu, Qun Huang, Xue‐Mei Sun, De‐Zhi Mu, Xi‐Hong Li

**Affiliations:** ^1^ Department of Pediatrics West China Second University Hospital Sichuan University Chengdu China; ^2^ Key Laboratory of Birth Defects and Related Diseases of Women and Children (Sichuan University) Ministry of Education Chengdu China; ^3^ Clinical Medical College Xiamen University Xiamen China

**Keywords:** apoptosis, autophagy, immunity‐related GTPase M1, p38 mitogen‐activated protein kinase, sepsis‐related encephalopathy

## Abstract

**Aims:**

Sepsis‐associated encephalopathy (SAE) is a common complication of severe sepsis. Our goal was to investigate the role of immunity‐related GTPase M1 (IRGM1) in SAE and its underlying mechanism.

**Methods:**

A mouse sepsis model was established by cecal ligation and perforation. SAE was diagnosed by behavior, electroencephalography, and somatosensory evoked potentials. Wild‐type mice with SAE were treated with SB203580 to block the p38 mitogen‐activated protein kinase (MAPK) signaling pathway. We assessed hippocampal histological changes and the expression of IRGM1, interferon‐γ (IFN‐γ), and p38 MAPK signaling pathway‐related proteins.

**Results:**

Immunity‐related GTPase M1 and IFN‐γ levels increased in the hippocampus, with apoptosis, autophagy, and the p38 MAPK signaling pathway activated in neurons. Administration of SB203580 to mice with SAE reduced apoptosis and autophagy. Relative to wild‐type mice with SAE, the general condition of *Irgm1^‐/‐^* mice with SAE was worsened, the p38 MAPK signaling pathway was inhibited, and neuronal apoptosis and autophagy were reduced. The absence of IRGM1 exacerbated SAE, with higher p38 MAPK signaling pathway activity and increased apoptosis and autophagy.

**Conclusions:**

During SAE, IRGM1 can at least partially regulate apoptosis and autophagy in hippocampal neurons through the p38 MAPK signaling pathway.

**Abbreviations**
Bax: Bcl‐2 associated X
Bcl‐2: B cell lymphoma‐2
CLP: cecal ligation and perforation
EEG: electroencephalography
HE: hematoxylin‐eosin
HR: heart rate
IFN: interferon
IRGM1: immunity‐related GTPase M1
*Irgm1^‐/‐^*: IRGM1 knockout
LC3: microtubule‐associated protein light chain 3
MAP: mean arterial pressure
MAPK: mitogen‐activated protein kinase
MK: MAKPAPK
NeuN: neuron‐specific nuclear (also known as Fox‐3, Rbfox3, and hexaribonucleotide binding protein‐3
p‐MK: phospho‐MAKPAPK
SAE: sepsis‐related encephalopathy
SEM: standard error
SEP: somatosensory evoked potential
SQSTM 1: sequestosome 1
TEM: transmission electron microscopy
TUNEL: TdT‐mediated DUTP nick‐end labeling

## 1 INTRODUCTION

Sepsis is a common critical illness in pediatric intensive care units, with associated brain injury in over 50% of septic patients.^1^ The primary clinical manifestation of sepsis‐associated encephalopathy (SAE) is cognitive impairment of varying severity, which is closely correlated with patient prognosis.^2^, ^3^, ^4^ Approximately 70% of septic patients enter comas and more than 80% have electroencephalographic abnormalities.^5^ The pathogenetic features of SAE include bacterial endotoxins, changes in blood‐brain barrier permeability, oxidative stress, and direct neuronal damage. The inflammatory cascade caused by bacterial invasion is an important pathogenic aspect of SAE.^4^, ^6^, ^7^

The interferon (IFN)‐inducible immunity‐related GTPase M1 (IRGM1) protein, previously characterized as an effector required for macrophage microbicidal activity, has been shown to regulate IFN‐γ–dependent host defense, lymphocyte survival, and autophagy.^8^, ^9^, ^10^, ^11^ A clinical study showed that the homozygous TT genotype at the *IRGM* (+313) rs10065172 locus is associated with reduced expression of IRGM in severe sepsis and higher mortality.^12^ In an experimental stroke study in mice, IRGM1 effectively activated autophagy in early stages, protected neurons from death in the ischemic area, and promoted apoptosis in the penumbra.^13^ Therefore, we hypothesized that IRGM1 is involved in the pathogenesis of SAE.

Apoptosis and autophagy are two common avenues to cell death,^14^ but there are few studies that investigate their role in SAE. Apoptosis plays a regulatory role in the heart, lungs, and liver during sepsis.^15^, ^16^, ^17^ Autophagy has protective effects in the myocardium, proximal renal tubules, and lungs.^18^, ^19^, ^20^ Our previous studies showed apoptosis and autophagy in the hippocampus during SAE, but the relevant regulatory mechanisms are not fully understood.^21^, ^22^ Others have reported that IFN can regulate apoptosis and autophagy by inducing the expression of IRGM1.^23^

In this study, we established a SAE model through cecal ligation and puncture (CLP) in wild‐type and IRGM1 knockout (*Irgm1* ^‐/‐^) mice to study the role of IRGM1 on neuronal apoptosis and autophagy in SAE.

## 2 MATERIALS AND METHODS

### 2.1 Animals

All experiments were designed and executed in accordance with the guidelines of the Research Animal Care Committee of Sichuan University. The suffering of experimental animals was alleviated whenever possible. Thirty‐day‐old, 18‐ to 22‐g (equivalent to human adolescents) male C57BL/6 wild‐type mice were purchased from Chengdu Dashuo Experimental Animals; *Irgm1* ^‐/‐^ mice were purchased from Cyagen Biosciences.^24^, ^25^ All mice had ad libitum food and water. Mice were housed at 22‐25°C and 55%‐58% relative humidity on a 12‐hour light/dark cycle.

### 2.2 Experimental groups and assays for neurological function

Mice were divided into sham operation control and experimental model groups: wild‐type sham operation (WTS), wild‐type model (WTM), wild‐type model intervention (WTMI), wild‐type model intervention control (WTMIC), gene knockout sham operation (GKS), and gene knockout model (GKM). Sepsis in the WTM, WTMI, WTMIC, and GKM groups was established by CLP.^26^ After mice were deeply anesthetized by inhalation isoflurane in 100% O_2_, the cecum was dissociated, ligated, and then punctured twice with an 18‐gauge needle. After a small amount of feces was extracted, the cecum was restored and the abdominal cavity sutured layer by layer. Immediately after surgery, Ringer solution (50 mL/kg) was intraperitoneally injected to protect against shock. Clindamycin and ceftriaxone (150 and 50 mg/kg, respectively) were intraperitoneally injected every 6 hours. For the sham operation groups, only the cecum was turned over after laparotomy and the abdomen was closed immediately without any other treatment.

Neurobehavioral, EEG, and somatosensory evoked potential (SEP) data were collected and evaluated in each group (n = 30) at 6 hours after surgery. Criteria for SAE diagnosis were neurobehavioral scores <6, accompanied by abnormal EEG and SEP.^27^, ^28^ Mice in WTS group and GKS group did not show SAE, and 30 mice in each group were included in the next experiment. In mice that received CLP, the incidence of SAE in mice in WTM, WTMIC, WTMI, and GKM groups was 26, 25, 23, and 28, respectively, and only those diagnosed with SAE were included in subsequent experiments. Mice in the WTMI group were intraperitoneally injected immediately following surgery with SB203580 (1 mg/kg, Enzo Life, dissolved in 1% DMSO).^29^, ^30^ Mice in the control WTMIC group were intraperitoneally injected with the same volume of 1% DMSO lacking SB203580.

### 2.3 Electroencephalography and somatosensory evoked potential

At 10 d before CLP, permanent epidural electrodes were implanted.^27^ After mice were anesthetized as described above, they were placed in a stereotactic apparatus (Model 963, Ultra Precise Small Animal Stereotaxic, David Kopf Instruments), with saline injections to maintain fluid balance. During implantation, body temperature, respiratory rate, heart rate, inhaled/expired CO_2_, and SpO_2_ were continuously monitored. Anesthesia depth was evaluated regularly. A 1.5‐cm incision was made along the mid‐sagittal plane of the skull, and periperiosteal soft tissue was removed. Three small, wired stainless‐steel screws were implanted epidurally, including a cortical (S1: 2.5 mm caudal to bregma, 2.5 mm right from midline) and left/right frontal (10 mm rostral to bregma, 1 mm lateral from midline) electrodes. Electrodes were wired to an 8‐pin receptacle (Mecap Preci‐Dip 917‐93‐108‐41‐005, Preci‐Dip Durtal SA, Delémont, Switzerland) and fixed to the skull with antibiotic bone cement (Simplex^™^ P bone cement with tobramycin, Stryker Nederland BV). The skin was stitched in a single layer around the receptacle. Two SEP‐evoking stimuli electrodes were positioned on the left lateral part of the tail base, spaced 3 mm from each other. EEGs recorded delta waves (0.5 ~ 3 Hz), theta waves (4 ~ 8 Hz), alpha waves (8 ~ 13 Hz), and beta waves (13 ~ 30 Hz). SEPs were elicited by multiple square‐wave pulses of 2‐ms duration, stimulus intensity of 5‐15 mA, wave width of 10 ms, and frequency of 3 Hz, generated with a Grass stimulator (Model S‐88, Grass Medical Instruments). P1 and N1 waves were recorded.

### 2.4 Vital signs

Mice were continuously anesthetized by inhalation isoflurane in 100% O_2_. An indwelling tube was inserted into the femoral artery and connected to a biological signal recorder (iWorx Systems, Inc). Mean arterial pressure (MAP) and heart rate (HR) were continuously monitored.

### 2.5 Neurobehavioral assays

Six hours after CLP, activity level, wound healing, infection, and other general conditions were observed and recorded. At the same time, a neurobehavioral test was performed.^28^ Pinnal, corneal, and tail‐flection reflexes were tested for simple nonpostural somatomotor function. Righting reflexes and escape responses were tested for evaluation of complex postural somatomotor function. Reflex scores were 0 (no response), 1 (reflex after >10 seconds), and 2 (normal). The highest possible score for the combined neurobehavioral tests was 10.

### 2.6 Sample collection

Mice were deeply anesthetized, and complete brains were excised with brain tissue remaining only on the coronal plane, starting at the optic chiasm and going back 1 cm. Brain samples from each group were randomly divided into three subgroups, with 5 mice in each. In the first, the hippocampus was dissociated and stored at −80°C for Western blotting. In the second, brain tissue was fixed in 4% paraformaldehyde (PFA) for hematoxylin‐eosin (HE), immunofluorescence, and TdT‐mediated DUTP nick‐end labeling (TUNEL) staining. In the third, the brain tissue was fixed in 2.5% Gluta electron microscopy fixative (Solarbio) for transmission electron microscopy (TEM).

### 2.7 HE staining

Brain tissue fixed in 4% PFA was dehydrated with alcohol, cleared with xylene, and embedded with paraffin. The sample was cut into 8‐μm sections and dried. Xylene was dewaxed, sections were rehydrated through an alcohol series, and staining was performed. Sections were scored for pathological changes by light microscopy (Olympus,). Three hippocampal fields from each mouse were randomly selected, and neurons were counted using Image J (NIH, Bethesda).

### 2.8 Transmission electron microscopy

A 1‐mm^3^ hippocampal sample was cut from the fixed tissue, rinsed with phosphate‐buffered saline (PBS, pH 7.3), and then fixed with 3% glutaraldehyde and 1% osmium tetroxide for 2 hours. The sample was embedded in epoxy resin, cut into 0.12‐μm sections, and then stained with 1% uranium acetate and 0.2% lead citrate. TEM was performed with an HT7700 120KV (Hitachi).

### 2.9 Western blotting

Hippocampal samples were deposited in ice‐cold lysis buffer composed of 50 mmol/L Tris‐HCl pH 7.4, 150 mmol/L NaCl, 10 mg/L NP‐40, and 0.1% protease inhibitor cocktail (Roche). Samples were homogenized on ice, centrifuged at 12 000 *g* for 30 minutes at 4°C, and then placed on ice for 30 minutes. Supernatants were harvested, and protein was quantitated using the BAC assay (Sigma‐Aldrich) to ensure consistent loading. Twenty µg of protein was separated on 12 or 15% gels and transferred to polyvinylidene difluoride membranes (Millipore). Blots were blocked with 2.5% nonfat milk in TBS‐T (10 mmol/L Tris‐HCl pH 8, 150 mmol/L NaCl, 0.05% (v/v) Tween 20) at room temperature for 1 hour and incubated with primary antibodies at 4°C overnight. Primary antibodies against rabbit anticleaved caspase‐3 (1:500, Cell Signaling Technology), mouse anti‐B cell lymphoma‐2 (Bcl‐2) (1:500, R&D Systems), and rabbit anti‐Bcl‐2 associated X (Bax) (monoclonal, 1:1000, Cell Signaling Technology) were used to detect apoptosis; rabbit anti‐microtubule‐associated protein light chain 3 (LC3) (1:1000, Abcam) and mouse anti‐sequestosome 1 (SQSTM1)/p62 (1:1000, Abcam) were used to detect autophagy, rabbit anti‐IRGM1 (1:500, Abcam), rabbit anti‐Interferon gamma (IFN‐γ) (1:1000, Abcam), rabbit anti‐MK 2 (1:1000, Abcam), rabbit anti‐phospho‐MAKPAPK (p‐MK) 2 (1:1000, Abcam), mouse anti‐GAPDH (monoclonal, 1:5000, loading control, ZSGB‐BIO), and mouse anti‐β‐tubulin (1:5000, loading control, ZSGB‐BIO). Blots were washed with TBS‐T 3 times for 10 minutes each and then incubated with a 1:5000 dilution of HRP‐conjugated anti‐rabbit or anti‐mouse secondary antibody (ZSGB‐BIO) at room temperature for 1 hour. Bands were visualized in the linear range with enhanced chemiluminescence (ECL, Millipore) using a gel imaging system (Bio‐Rad). All bands were quantitated using Image J, and relative intensities of each target protein band against GAPDH or β‐tubulin controls were calculated.

### 2.10 Immunofluorescence staining

Brain tissue fixed in 4% PFA was embedded in 2.5% agarose and cut into 40‐μm sections by an oscillating slicer. Agarose on the sections was removed and washed with PBS for 3 times, 5 minutes per wash. Sections were then incubated in 0.3% Triton X‐100 and blocking reagent (3% serum protein, 2% fresh bovine serum, 0.2% Triton X‐100) for 30 minutes and 1 hour, respectively, and then incubated at 4 ℃ overnight with primary antibody: rabbit anti‐IRGM1 polyclonal (1:200) or mouse anti‐neuron‐specific nuclear (NeuN) monoclonal (1:500, Abcam). Sections were washed 3 times with PBS and incubated with DyLight 488‐conjugated donkey anti‐rabbit IgG (1:500, Jackson ImmunoResearch) or Cy3‐conjugated donkey anti‐mouse IgG (1:500, Jackson ImmunoResearch) in the dark for 2 hours at room temperature. After washing 3 times with PBS, nuclei were stained in the dark with 4, 6‐diamidino‐2‐phenylindole (DAPI, 1:500, Beyotime) for 10 minutes at room temperature. Finally, the sections were flattened in PBS and attached to glass slides, treated with antifluorescence quenching agent (Beyotime), and covered with coverslips. A laser scanning confocal microscope (Olympus) and FV10‐ASW‐4.2 software (Olympus) were used to image the sections. Three fields from the dentate gyrus of each animal were randomly selected for quantitation of the expression of IRGM1 using ImageJ.

### 2.11 TUNEL staining

Brain tissues were cut into 40‐μm sections and then stained according to manufacturer's instructions with the DeadEnd^™^ Fluorometric TUNEL System (Promega). Finally, sections were washed with PBS and counterstained with DAPI (1:500) for 5 minutes in the dark at room temperature. Sections were mounted onto glass slides and imaged by confocal laser scanning microscopy as described above. Three fields from dentate gyrus for each animal were randomly selected for scoring of TUNEL‐positive cells and the total number of nuclei using ImageJ.

### 2.12 Statistical analyses

Data are presented from 3 independent tests, n = 5 for each group, and expressed as means ± standard error (SEM). Statistical analyses were performed using Statistical Package for Social Sciences (version 19.0, Chicago, IL, USA) software. Neurobehavioral scores were analyzed by Friedman repeated measures ANOVA. Statistical significance between groups was analyzed by one‐way ANOVA followed by the Student‐Newman‐Keuls test. *P* < .05 was regarded as the threshold of statistical significance.

## 3 RESULTS

### 3.1 CLP mice have abnormal behavior, irregular EEG and SEP, and deteriorating vital signs

At 6 hours after modeling, we collected and analyzed the neurobehavioral scores, vital signs, EEG, and SEP of each group of mice (Table 1). Compared with the sham operation group, the neurobehavioral scores of mice in the CLP group were significantly decreased. Scores in the GKM group were decreased relative to the WTM group. Similarly, compared with the sham operation group, the vital signs of the CLP group were worse, showing decreased MAP and increased HR. The deterioration of GKM group was more severe than that of the WTM group.

**Table 1:** Neurobehavioral test scores, vital signs, EEG, and SEP at 6 h postsurgery

Group				
	WTS	WTM	GKS	GKM
Neurobehavioral test scores	9.80 ± 0.447	5.20 ± 1.10*	9.60 ± 0.547	4.80 ± 0.447**
MAP (mm Hg)	92.4 ± 3.22	68.3 ± 3.92*	88.3 ± 4.02	54.6 ± 2.88** ^,^ ***
HR (beats/min)	318 ± 18.3	404 ± 26.9*	344 ± 33.2	469 ± 24.3** ^,^ ***
EEG				
Alpha wave frequency (%)	27.4 ± 1.09	17.2 ± 1.48*	24.3 ± 2.52	12.4 ± 2.03** ^,^ ***
Delta wave frequency (%)	13.9 ± 1.92	21.2 ± 2.28*	15.9 ± 3.08	27.3 ± 3.10** ^,^ ***
Beta wave frequency (%)	42.4 ± 2.83	38.4 ± 2.91*	42.0 ± 3.01	34.3 ± 3.32** ^,^ ***
Theta wave frequency (%)	24.9 ± 1.02	18.3 ± 2.19*	23.7 ± 2.30	16.5 ± 2.54**
SEP				
P1 amplitude (μV)	16.4 ± 2.27	15.2 ± 2.13	16.3 ± 2.29	14.3 ± 2.93
P1 latency (ms)	17.4 ± 3.20	24.2 ± 2.39*	18.8 ± 4.94	27.9 ± 3.28**
N1 latency (ms)	27.5 ± 3.02	30.2 ± 2.49	29.4 ± 3.56	32.1 ± 2.58

Note

Data are means ± SEM, n = 5 for each group.

* *P* < .05 vs WTS.

** *P* < .05 vs GKS.

*** *P* < .05 vs WTM.

Mice in the CLP group showed significantly irregular EEGs relative to the sham controls (Table 1), including decreased frequency of alpha and theta waves and increased frequency of delta and beta waves. The EEG of GKM group was more irregular than those of the WTM group. SEP showed no significant change at 6 hours after surgery, and only P1 latency was observed after CLP compared with the sham control group.

Subsequently, as shown in Table 1, mice undergoing CLP were evaluated at 6 hour after surgery, and SAE was diagnosed as described.^27^, ^28^ Only mice diagnosed with SAE in the WTM, GKM, and GKMI groups were used in subsequent experiments. No mice in the WTS and GKS groups were diagnosed with SAE; the incidence of SAE in the WTM and the GKM groups was 86.7% (26/30) and 93.3% (28/30), respectively.

### 3.2 Activation of apoptosis in hippocampus of SAE mice

**Figure 1:**
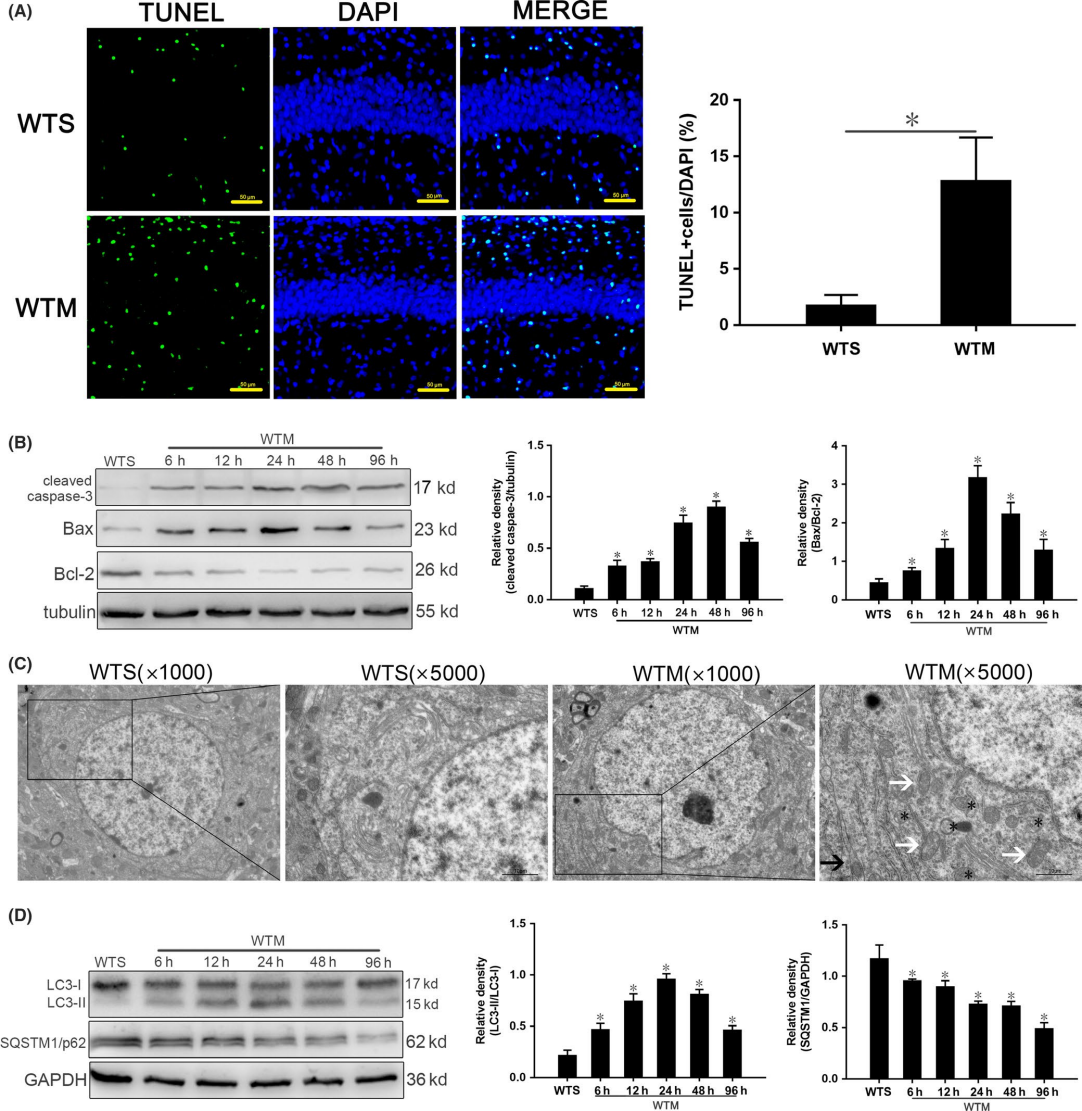
Apoptosis and autophagy are activated in hippocampus of SAE mice. A, TUNEL‐positive cells at 24 h. Apoptotic cell bodies are stained green, and cell nuclei are stained blue. Scale bar = 50 μm. B, Expression of cleaved caspase‐3, Bcl‐2 and Bax quantitated by Western blotting. Protein levels were normalized to those of β‐tubulin and are shown as relative arbitrary units. C, TEM showing increased autophagosomes (*), expanded endoplasmic reticulum (black arrows), and swelled mitochondria (white arrows) in WTS mice. Scale bar = 10 μm. D, Expression of LC3 and SQSTM1/p62 quantitated by Western blotting. The protein levels were normalized to those of GAPDH and are shown as relative arbitrary units. Data are from 3 independent tests, n = 5 for each group, *P < .05

To observe hippocampal apoptosis, we counted proportions of TUNEL‐positive cells (defined by costaining of apoptotic bodies and nuclei). Proportions in the WTM group were significantly higher than those in the WTS control group (Figure 1A). Meanwhile, expression of cleaved caspase‐3 and Bax/Bcl‐2 in hippocampus of the WTM group was significantly higher than those of the WTS controls each time point tested (Figure 1B).

### 3.3 Activation of autophagy in hippocampus of SAE mice

Autophagy in hippocampal neurons at 24 hours after surgery was observed using TEM. Compared with the WTS group, neurons in the WTM group exhibited more autophagosomes, expanded endoplasmic reticulum, and swollen mitochondria (Figure 1C).

SAE altered the expression of two autophagy proteins: LC3, involved in multiple aspects of autophagic activity, and SQSTM1, a cargo receptor that sequesters and mediates the delivery of aberrant proteins. Compared with WTS controls, LC3‐II/I in the WTM group increased gradually from 6 hours to a peak at 24 hours and then decreased (Figure 1D); however, SQSTM1/p62 expression decreased gradually, with expression at all time points significantly lower than that in the WTS group (Figure 1D).

### 3.4 SAE increases hippocampal IRGM1 and IFN‐γ expression

**Figure 2:**
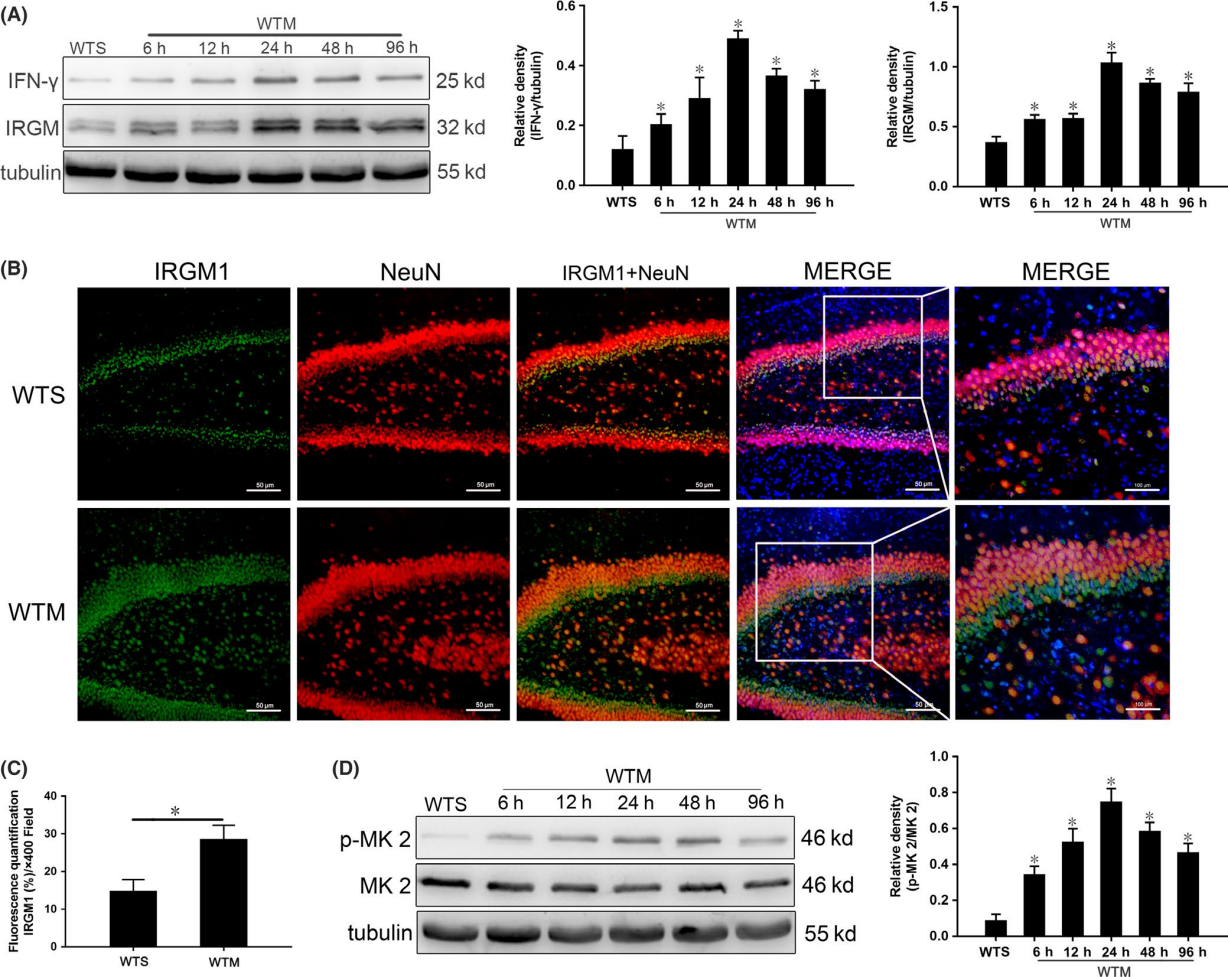
Increased hippocampal expression of IFN‐γ and IRGM1 and activation of the p38 MAPK signaling pathway in SAE. A, Expression of IFN‐γ and IRGM1 quantitated by Western blotting. Protein levels are normalized to those of β‐tubulin and shown as relative arbitrary units. B, Immunofluorescent localization of IRGM1 (green), NeuN (red), and nuclei (blue); scale bar = 50 or 100 μm. C, Semi‐quantitative analysis of IRGM1 expression from immunofluorescence images. D, Expression of p‐MK 2 and MK 2 quantitated by Western blotting. Protein levels are normalized to those of β‐tubulin and shown as relative arbitrary units. Data are from 3 independent tests, n = 5 for each group, *P < .05

Western blotting showed that the expression of IRGM1 and IFN‐γ in the WTM group was higher at each time point compared with WTS controls (Figure 2A). We further observed by immunofluorescence that IRGM1 expression in the WTM group was significantly higher than that of WTS controls at 24 hours (Figure 2B‐C).

### 3.5 The p38 MAPK signaling pathway is activated in SAE

To investigate the role of the p38 MAPK signaling pathway in SAE mice, we measured the expression of MK 2 and p‐MK 2 in hippocampus. MK 2 is the direct downstream target of p38 MAPK. Expression of p‐MK 2/MK 2 in the WTM group was increased at all time points compared with those in WTS controls, reaching a maximum value at 24 hours (Figure 2D).

### 3.6 Inhibition of the p38 MAPK pathway reduces apoptosis and autophagy

To further test whether apoptosis and autophagy of hippocampal neurons are regulated by the p38 MAPK signaling pathway in SAE, SB203580, a specific inhibitor of phosphorylation of MK 2,^29^, ^30^ was used for further intervention at 24 hours post‐CLP surgery.

**Figure 3:**
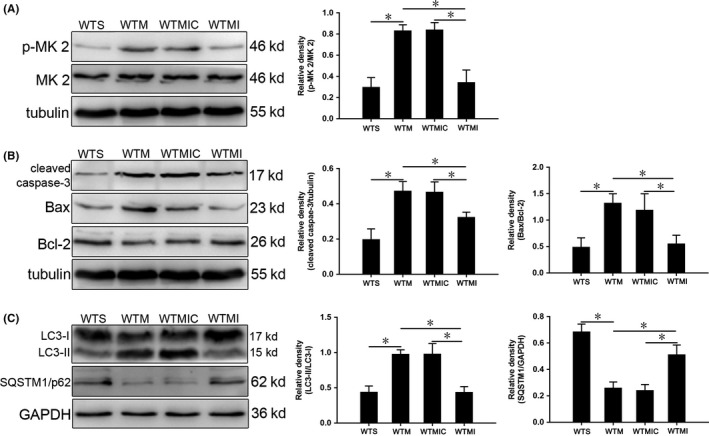
SB203580 inhibits p38 MAPK signaling, apoptosis, and autophagy in hippocampus of SAE mice at 24 h. A, Expression of p‐MK 2 and MK 2. B, Expression of cleaved caspase‐3, Bax, and Bcl‐2. C, Expression of LC3 and SQSTM1/p62 quantitated by Western blotting. Protein levels are normalized to those of GAPDH or β‐tubulin and shown as relative arbitrary units. Data are from 3 independent tests, n = 5 for each group, *P < .05

First, we found that expression of p‐MK 2/MK 2 was significantly reduced in the WTMI group relative to the WTM and WTMIC control groups (Figure 3A). Meanwhile, expression of the apoptosis‐related proteins cleaved caspase‐3 and Bax/Bcl‐2 in the WTMI group was significantly decreased compared with those in WTM and WTMIC controls (Figure 3B). In addition, expression of the autophagy‐related protein LC3 II/I was decreased and SQSTM1/p62 was increased (Figure 3C).

### 3.7 Expression of IFN‐γ is not significantly changed in *Irgm1* ^‐/‐^ mice with SAE

**Figure 4:**
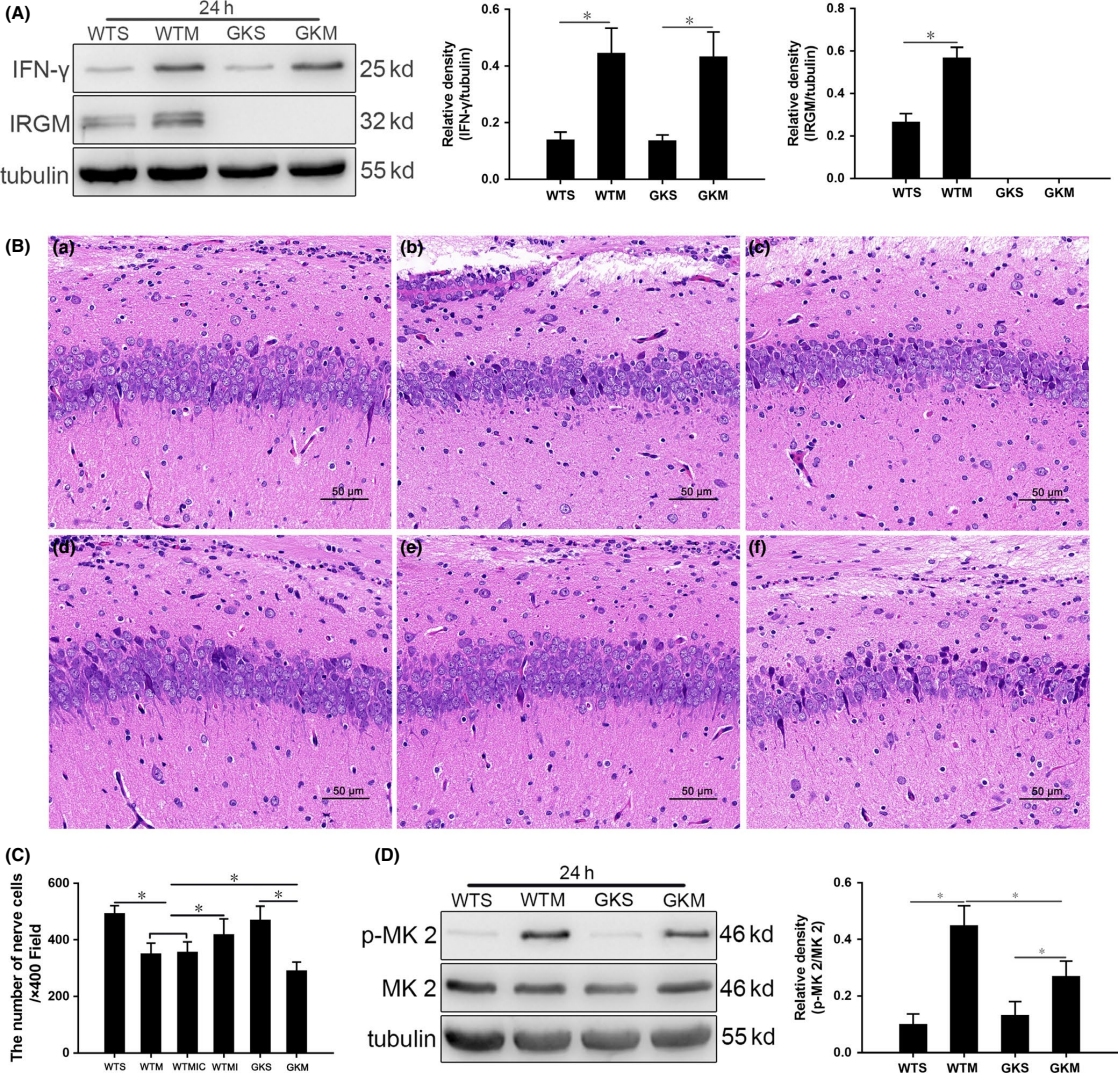
Pathological changes in the hippocampus of mice with SAE are aggravated, and p38 MAPK signaling is inhibited in IRGM1 knockout mice. A, Expression of IFN‐γ and IRGM1 in the hippocampus at 24 h quantitated by Western blotting. Protein levels are normalized to those of β‐tubulin and are shown as relative arbitrary units. B, Pathological changes in the hippocampus of mice 24 h postsurgery. (a) Relatively normal morphology in the WTS group; in (b) WTM and (c) WTMIC groups, some cells dissolve and numbers of cells decrease; (d) in the WTMI group, cells are irregular in shape and somewhat disordered in arrangement; (e) in the GKS group, morphology is relatively normal; and (f) in the GKM group, cells are reduced and disordered, presenting as acute traumatic changes. Scale bar = 50 μm. C, Cell counts from HE‐stained sections. D, Expression of p‐MK 2 and MK 2 at 24 h quantitated by Western blotting. Protein levels are normalized to those of β‐tubulin and shown as relative arbitrary units. Data are from 3 independent tests, n = 5 for each group, *P < .05

To test for a regulatory relationship between IFN‐γ and IRGM1, and to confirm the knockout of *Irgm*, we measured IFN‐γ and IRGM1 expression in the hippocampus at 24 hours. IFN‐γ was lower in the WTS and GKS groups than in SAE mice, but there was no difference between groups. Moreover, IFN‐γ expression was higher in the WTM and GKM groups than in sham controls (Figure 4A), but there was no difference between the experimental groups. Expression of IRGM1 in the WTM group was higher than that in the WTS group (Figure 4A); however, expression was not observed in the GKS and GKM groups.

### 3.8 IRGM1 protects against pathological changes in SAE

We observed hippocampal histological changes in mice with SAE, with the greatest difference between groups at 24 hours postsurgery. As shown in Figure 4B‐C, hippocampal cells were abundant and normally arranged in both the WTS (a) and GKS (e) groups. However, in the WTM (b) and WTMIC (c) groups, some cells were damaged in the hippocampus, with the number of cells significantly reduced. After SB302580 treatment, cells in the WTMI (d) group were more orderly and significantly more numerous than those of the WTM and WTMIC groups. In the GKM (f) group, cell numbers were decreased those in the WTM, WTMIC, and GKS groups, and they were disordered, leading to the disruption of normal hippocampal structure.

### 3.9 The p38 MAPK signaling pathway is inhibited in *Irgm1* ^‐/‐^ mice with SAE

Since we observed inhibition of the p38 MAPK signaling pathway (Figure 3A), to further study the interaction between IRGM1, the p38 MAPK signaling pathway, apoptosis, and autophagy, we established the SAE model in *Irgm1* ^‐/‐^ mice.

The p‐MK 2/MK 2 expression levels were lowest in the WTS and GKS group, with slightly higher expression in GKM group, but levels measured in the GKM group were significantly lower than those in the WTM group (Figure 4D).

### 3.10 SAE‐induced apoptosis is reduced in *Irgm1* ^‐/‐^ mice

**Figure 5:**
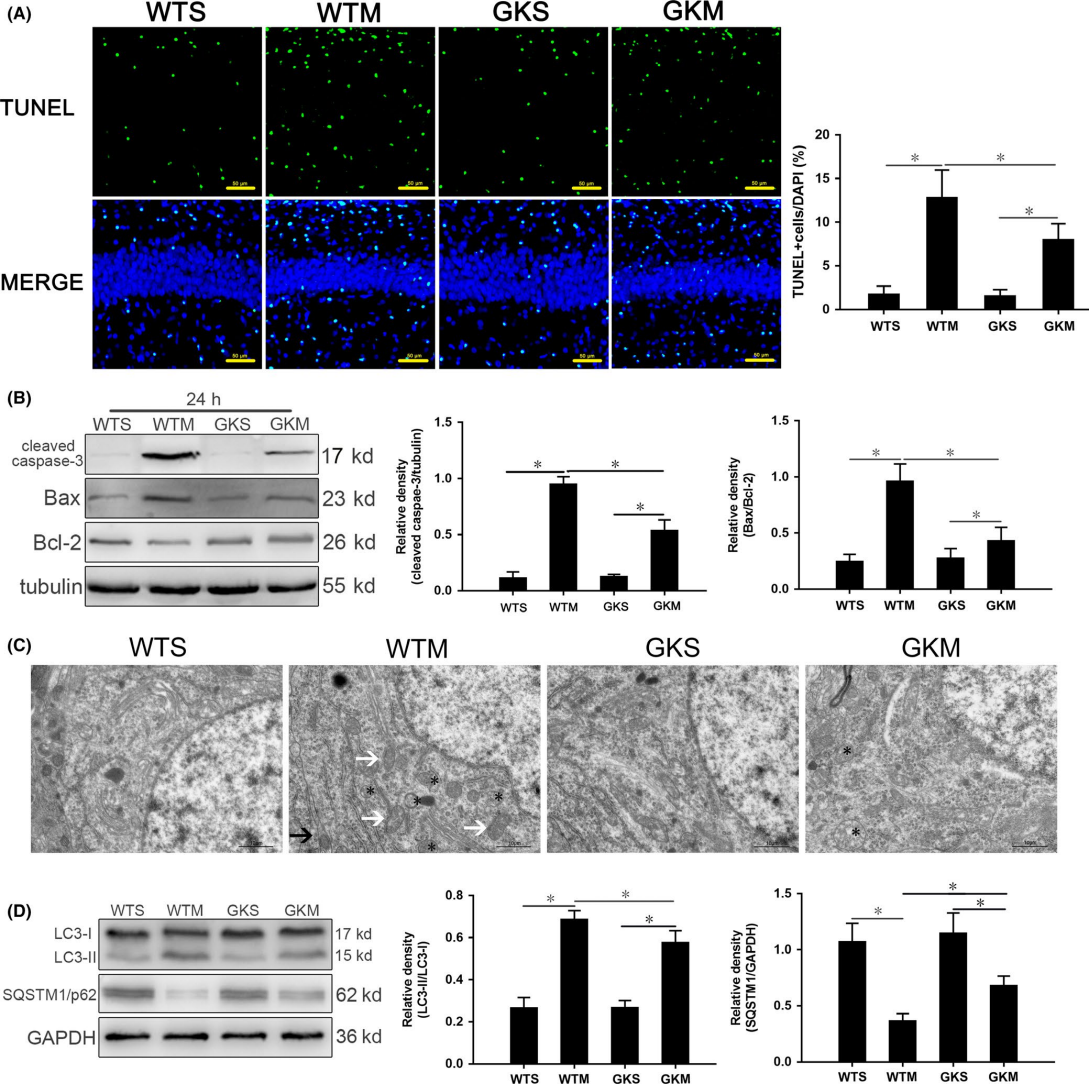
The absence of IRGM1 reduced apoptosis and autophagy in mice with SAE. A, TUNEL staining; apoptotic bodies are green, and nuclei are blue. Scale bar = 50 μm. B, Expression of cleaved caspase‐3, Bcl‐2, and Bax quantitated by Western blotting. Protein levels are normalized to those of β‐tubulin and shown as relative arbitrary units. C, TEM showing lack of autophagosomes in the WTS and GKM groups; in the WTM group, there were many autophagosomes enclosing damaged organelles and proteins (*); and in the GKM group, there were a few autophagosomes (*) and no complete intracellular structures were observed. Scale bar = 10 μm. D, Expression of LC3 and SQSTM1/p62 quantitated by Western blotting. Protein levels are normalized to those of GAPDH and shown as relative arbitrary units. Data are from 3 independent tests, n = 5 for each group, *P < .05

As described above, the difference between WTS group and WTM group was the largest at 24 hours postsurgery. Thus, we further measured the effect of IRGM1 on neuronal apoptosis using TUNEL staining and Western blotting to quantitate the expression of apoptosis proteins at 24 hours. We found no significant difference in the proportions of TUNEL‐positive cells between the WTS and GKS groups (Figure 5A). The proportion in the GKM group was higher than that in the sham control group, but lower than that in the WTM group. Similarly, the expression levels of cleaved caspase‐3 and Bax/Bcl‐2 were lower in the GKM group relative to the sham and WTM groups (Figure 5B).

### 3.11 Reduced activation of autophagy in hippocampal neurons of *Irgm1* ^‐/‐^ mice

The 24‐hour time point was selected to further analyze the influence of IRGM1 on autophagy. As shown in Figure 5C, we found that hippocampal neurons in the WTS and GKS groups were ultrastructurally normal, while those in the GKM group were more disorganized than those in the WTM group, with severe organelle destruction and fewer autophagic vesicles. We found that expression of LC3‐II/I in the hippocampus of the GKM group decreased, while expression of SQSTM1/p62 increased, relative to the WTM group (Figure 5D).

## 4 DISCUSSION

We found that in hippocampus of mice with experimental SAE induced by CLP, expression of IFN‐γ and IRGM1 was increased, with the increase in IRGM1 to some extent regulating apoptosis and autophagy through the p38 MAPK signaling pathway. Moreover, IRGM1 had a protective effect against SAE, but the mechanism is unclear with respect to apoptosis and autophagy.

Mice were evaluated at 6 hours after CLP; compared with the WTM group, the GKM group showed abnormal behavior and poorer vital signs, with irregular EEG and SEP (Table 1). We also found that the pathological changes in the hippocampus of *Irgm1^‐/‐^* mice with SAE were more severe than those of wild‐type mice with SAE (Figure 4B). Therefore, we conclude that IRGM1 may be one of the important protective factors for SAE.

There are many ways in which cell death can be initiated, with apoptosis and autophagy the dominant types.^14^ These mechanisms all share a mitochondria‐centric regulatory mechanism.^31^ With the transmission of a cascade of pro‐apoptotic signals, cells can initiate anti‐apoptotic signaling mechanisms in response to stress. When pro‐apoptotic signals prevail, the mitochondrial membrane begins to become more permeable as a consequence of the action of the pro‐apoptotic protein Bax.^32^, ^33^ We observed a significant increase in the proportion of TUNEL‐positive cells in the hippocampus of SAE mice. Moreover, expression of cleaved caspase‐3 and Bax/Bcl‐2 was increased over the sham group at all time points (Figure 1A‐B).

Autophagy is accompanied by a large amount of cytoplasmic vacuolation.^34^ The process consists of vesicles encapsulating damaged substances, fusing with lysosomes to form autophagosomes, which are finally degraded. Autophagy is important for maintaining cell homeostasis, but excessive autophagy is detrimental to survival.^35^ During autophagy, LC3‐I in the cytoplasm is transformed into LC3‐II on the autophagosomal membrane, while SQSTM1/p62 is the ubiquitin‐binding substrate to bind to the autophagosome.^36^ We found more double‐membrane autophagosomes in hippocampal neurons of SAE mice (Figure 1C). At each time point after inducing SAE, expression of LC3‐II/LC3‐I was significantly increased over sham controls, while expression of SQSTM1/p62 gradually decreased (Figure 1D), indicating activation of autophagy.

An increasing number of studies have confirmed that IFN‐γ plays a key role in autophagy induced by IRGM, an important aspect of pathogen resistance.^37^ When IRGM expression increased, autophagy was enhanced significantly, improving cell survival.^38^ In this study, expression of IFN‐γ and IRGM1 in the hippocampus of wild‐type SAE mice was significantly increased over sham controls (Figure 2A‐C). We conclude that there may be a functional correlation between the concomitant increases.

To find a link between increased IRGM1 and activation of apoptosis and autophagy, we selected the p38 MAPK signaling pathway,^39^, ^40^ one of the most important in mammalian cells.^41^ In both in vitro and in vivo experiments, activation of this pathway has been shown to effectively induce apoptosis and autophagy.^42^, ^43^, ^44^, ^45^ We found that p38 MAPK signaling was activated in SAE (Figure 2D). We then treated mice with the specific signaling pathway inhibitor SB203580, finding significant inhibition of the p38 MAPK signaling pathway in SAE mice, with apoptosis and autophagy of neurons also inhibited (Figure 3). Therefore, we conclude that neuronal apoptosis and autophagy may be regulated, at least in part, by the p38 MAPK signaling pathway.

Immunity‐related GTPase M1, a member of the immunity‐related GTPase family, is an IFN‐inducible intracellular protein found in the Golgi complex that has been implicated in a wide range of biological functions, including cell‐mediated immune responses and immunoregulation.^13^ To determine whether the regulation of IRGM1 on apoptosis and autophagy of neurons is also induced by IFN‐γ, we set up our SAE model in *Irgm1* ^‐/‐^ mice. We found that the expression of IFN‐γ was not significantly changed, suggesting that IFN‐γ may be upstream of IRGM1, in apoptosis and autophagy regulation (Figure 4A). This is similar to the data reported by Murray et al, who found that IFN‐γ induced expression of IRGM1 in *Leishmania* infection in liver.^46^

As shown in Figure 4D, we also found that the p38 MAPK signaling pathway in the hippocampus of *Irgm1^‐/‐^* mice was inhibited. Although p‐MK 2/MK 2 in GKM was reduced compared with WTM, it was still elevated in GKM relative to GKS. Thus, the absence of IRGM1 did not completely block the activation of p38 MAPK signaling induced by SAE, indicating that factors other than IRGM1 are likely involved in activation. Moreover, *Irgm^‐/‐^* mice with SAE were more abnormal than wild‐type controls behaviorally and histologically (Table 1 and Figure 4B), suggesting that IRGM1 still had a protective effect. Apoptosis and autophagy of neurons were also significantly reduced (Figure 5). The results of our study are similar to those reported by He et al^13^, who found that cerebral autophagy and apoptosis in *Irgm1^‐/‐^* mice with stroke were reduced.

Although our research has some important implications, there are some limitations. First, our 30‐day‐old mice are closest in physiological age to human adolescents, so it is unclear whether similar conclusions can be drawn for other age groups. In addition, whether the protective effect of IRGM1 on SAE is directly related to the induction of apoptosis and autophagy needs further study. In other experiments, increase in IRGM may promote^47^, ^48^ or inhibit apoptosis,^38^, ^49^ which may be related to differences in experimental subjects, disease models, or time points of intervention. It is important to note that over expression of autophagy factors can cause cell damage, so whether IRGM1‐induced autophagy directly protects neurons in this experiment is unknown. Finally, this study only showed that IFN‐γ is not regulated by IRGM1, but whether IRGM1 is regulated by IFN‐γ in the hippocampus of SAE mice is still not known, and whether p38 MAPK signaling plays a role in this should be a subject of further study, which will help us further clarify the mechanism of IRGM1's protective effect in SAE in mice.

In summary, this study suggests that the increase in IRGM1 has a protective effect on SAE mice and that it can also activate the p38 MAPK signaling pathway, which then induces apoptosis and autophagy of neurons in the hippocampus. Our experiment provides new possibilities for the prevention and treatment of SAE patients clinically, suggesting that IRGM1 is an effective target.

## CONFLICT OF INTEREST

There are no conflicts of interest to declare.
